# Hope at the end of life: Can hope endure when life nears its end?

**DOI:** 10.1017/S1478951525101089

**Published:** 2025-11-03

**Authors:** Jeff Clyde Corpuz

**Affiliations:** Department of Theology and Religious Education, College of Liberal Arts, De La Salle University, Manila, Philippines

What does it mean to hope when one stands at the threshold of death? This question captures one of the deepest human paradoxes. When physical decline is irreversible and medical care shifts from cure to comfort, hope does not disappear but transforms. It becomes less about survival and more about meaning, connection, and transcendence. In this transformation, hope affirms human dignity when life’s limits become clear. For patients nearing death, hope often reframes the question from “How long do I have?” to “What remains possible for me?”

## Hope and the palliative care context

Palliative and supportive care aim to improve quality of life for people with life-threatening illness by addressing physical, psychosocial, and spiritual suffering. The World Health Organization (2020) defines palliative care as an approach that enhances the well-being of both patients and caregivers. Approximately, 56.8 million people worldwide require palliative care each year, with 25.7 million in their final year of life (World Health Organization 2020).

Within this setting, hope becomes a clinical and existential phenomenon. Szabat and Knox (2021) identify three interwoven “shades” of hope in end-of-life care: the dynamic meaning of hope, the dialectic of hope and despair, and the transcendent dimension of hope. These elements frame hope as an active movement toward being, even in decline. Informed by Lear’s philosophical notion of “radical hope,” Frush (2025) emphasizes that in palliative contexts, hope persists as courage – the willingness to continue imagining meaning when certainty collapses.

Empirical studies reinforce this. Julião et al. (2021) found that documenting hope in patient records correlates with more holistic care and improved psychosocial outcomes. Beng et al. (2022) describe hope as an inner energy that sustains people through suffering and loss. Understanding how hope functions helps healthcare professionals create strategies that nurture it among patients approaching death. Kirby et al. (2021) further highlight that hope is collectively negotiated during family meetings, not a private emotion but a shared process of redefining goals and expectations.

## Existential and spiritual dimensions of hope

Recent work in supportive oncology and palliative medicine has widened the scope of care beyond symptom management to include meaning-making, spirituality, and existential well-being (Austin et al. 2025; Breitbart 2002). These dimensions are now viewed as essential to quality of life in terminal illness. Clinicians recognize that patients often draw strength from spiritual narratives or moral frameworks that allow them to reinterpret suffering as a space for reconciliation or personal transformation (Guedes et al. 2021).

Hope does not remove suffering but reshapes its significance (Breitbart 2003). It enables patients to endure with dignity and to integrate their illness into a coherent life story. For caregivers, hope mitigates compassion fatigue and moral distress. Maintaining professional hope – through reflection, community, or faith – supports resilience and compassionate presence. As Chapman and Komesaroff (2023) argue, what is needed is not nostalgia for lost normalcy but “radical hope,” a reorientation that embraces uncertainty as a site for moral growth and relational depth.

## Recent research and practical developments

Between 2020 and 2025, research on hope in palliative care evolved from conceptual exploration to empirical validation. The Herth Hope Index, long used to measure hope, was psychometrically validated across new cultural contexts (Herth and Sarasua 2022; Nikoloudi et al. 2021). Predictive models now integrate clinical, psychological, and spiritual factors to identify patients at risk of hopelessness, enabling early referral for psychosocial or spiritual support (Corpuz 2025).

Intervention research has expanded rapidly. Dignity Therapy and life-review interventions consistently improve hope levels and existential well-being (Julião et al. 2021). Mroz et al. (2025) examined the use of empathic self-disclosure by healthcare providers during Dignity Therapy. Their findings suggest that thoughtful self-disclosure strengthens therapeutic relationships and conveys authentic empathy. They also note the need for further research to assess both benefits and risks, recommending that Dignity Therapy training integrate guidance on appropriate self-disclosure.

Velić et al. (2023) propose that structured interventions engaging family and friends under professional guidance may further enhance hope, reinforcing its collective and relational dimensions. Similarly, Bertaud et al. (2025a) introduce the concept of “hope pluralism” in antenatal palliative care, emphasizing the coexistence of multiple and sometimes conflicting hopes among clinicians and families. Recognizing this pluralism allows healthcare professionals to approach hope not as a single emotion to be managed but as a dynamic dialogue between perspectives.

## Toward an integrative understanding of hope

The evolving scholarship points toward an integrative vision of hope. It encompasses emotional resilience, ethical discernment, relational empathy, and spiritual transcendence (Corpuz 2024). Hope becomes a therapeutic resource and an ethical stance. Its expression varies – from hope for cure to hope for comfort, peace, or reconciliation – but in each case, it sustains meaning, belonging, and connection. In a recent study, Paananen and Logren (2025) describe “palliative hope work” as the way caregivers help families of people with late-stage dementia hold on to meaning and peace amid loss. Through open and compassionate conversations, caregivers remind families that their loved ones are still present as persons, still cared for, and still capable of sharing moments of connection. By acknowledging both life and death with honesty and empathy, caregivers help families find acceptance and appreciation, supporting them to cherish what remains and prepare for a peaceful end with dignity (Paananen and Logren 2025).

Incorporating radical hope and hope pluralism into clinical practice invites a more inclusive understanding of human experience at the end of life. This approach resists reducing hope to measurable outcomes, acknowledging its philosophical, moral, and relational dimensions. It also calls for healthcare systems to support clinicians in cultivating their own hope, as they accompany patients through uncertainty and loss. For Bertaud et al. (2025b), the ethics of care fills key gaps in training by focusing on how practitioners relate to and engage with patients, families, colleagues, and their own inner lives. At its core, palliative care is built on relationships.

## Conclusion

Hope at the end of life is not the denial of death but the affirmation of life’s lasting worth. It transforms fear into peace, obligation into calling, and sorrow into gratitude. Within palliative and supportive care, hope serves as a sustaining force that preserves dignity and meaning when cure is beyond reach. It calls patients, families, and caregivers to live the present moment with courage and compassion. Though death closes the body’s journey, it does not silence the human spirit. Hope endures as a quiet strength, shifting from the desire for more time to the embrace of connection, comfort, and presence. For indeed, hope remains, even as life nears its end.

## Data Availability

The author has nothing to report.
